# Engineering Topological Surface State of Cr-doped Bi_2_Se_3_ under external electric field

**DOI:** 10.1038/srep43626

**Published:** 2017-03-08

**Authors:** Jian-Min Zhang, Ruqian Lian, Yanmin Yang, Guigui Xu, Kehua Zhong, Zhigao Huang

**Affiliations:** 1 Fujian Provincial Key Laboratory of Quantum Manipulation and New Energy Materials, College of Physics and Energy, Fujian Normal University, Fuzhou 350117, China; 2 Fujian Provincial Collaborative Innovation Center for Optoelectronic Semiconductors and Efficient Devices, Xiamen 361005, China

## Abstract

External electric field control of topological surface states (SSs) is significant for the next generation of condensed matter research and topological quantum devices. Here, we present a first-principles study of the SSs in the magnetic topological insulator (MTI) Cr-doped Bi_2_Se_3_ under external electric field. The charge transfer, electric potential, band structure and magnetism of the pure and Cr doped Bi_2_Se_3_ film have been investigated. It is found that the competition between charge transfer and spin-orbit coupling (SOC) will lead to an electrically tunable band gap in Bi_2_Se_3_ film under external electric field. As Cr atom doped, the charge transfer of Bi_2_Se_3_ film under external electric field obviously decreases. Remarkably, the band gap of Cr doped Bi_2_Se_3_ film can be greatly engineered by the external electric field due to its special band structure. Furthermore, magnetic coupling of Cr-doped Bi_2_Se_3_ could be even mediated via the control of electric field. It is demonstrated that external electric field plays an important role on the electronic and magnetic properties of Cr-doped Bi_2_Se_3_ film. Our results may promote the development of electronic and spintronic applications of magnetic topological insulator.

The interest of topological insulators (TIs) is to a large extent driven by the exotic surface electronic properties and the anticipated numerous potential applications in semiconductor spintronics^1^,^2^,^3^,^4^,^5^,^6^. The gapless topological surface states of TI, which is protected by time-reversal symmetry (TRS), also stimulate extensive theoretical and experimental research^7^,^8^,^9^,^10^,^11^,^12^,^13^,^14^,^15^. Incorporating magnetic ions into the host TI materials will break the TRS, opening a gap on the Dirac point. It is revealed to be an effective way to obtain the long-range magnetic order in TI^16^,^17^,^18^,^19^, especially in Bi_2_Se_3_^8^,^9^,^10^. Previous studies show that ferromagnetic (FM) ordering can be developed even in the insulating regime in Cr-doped Bi_2_Se_3_^20^,^21^,^22^,^23^,^24^. Remarkably, Cr-doped Bi_2_Se_3_ is promising for realizing many interesting quantum phenomena, such as topological magnetoelectric effect^25^ and the long-sought quantum anomalous Hall effect (QAHE)^21^,^26^,^27^,^28^.

The ability to modulate the band gap of Cr-doped Bi_2_Se_3_ could be significant for semiconductor spintronics and the application of TIs^12^,^29^. External electric field is revealed to have significant influence on bulk and SSs of Tis^30^,^31^,^32^,^33^,^34^,^35^,^36^,^37^,^38^,^39^,^40^,^41^. In particular, the magnetism of MTI can be external controlled by carrier-mediated Ruderman-Kittel-Kasuya-Yosida (RKKY) coupling^16^,^36^,^42^,^43^,^44^,^45^,^46^,^47^. Recent study revealed that the ultrathin Bi_2_Se_3_ films with a hybridization gap, which is induced by the coupling between two SSs, become gapless by the application of external electric field. This is due to the movement of electrons in the opposite direction of electric field^31^. Liu *et al*. also reports that external electric field can close or reopen a gap for surface states of Bi_2_Se_3_ film^32^. Wang *et al*. even revealed that electric field can induce a quantum phase transition in the magnetic topological insulators^45^. However, the mechanism of electronic manipulation by external electric field in pure Bi_2_Se_3_ is still inconsistent. The efficient electric-field control of the electronic and magnetic properties in insulating Cr-doped Bi_2_Se_3_ remains elusive.

In this paper, we performed a first-principles calculation within density functional theory (DFT) to investigate the pure and Cr-doped Bi_2_Se_3_ film under external electric field. We find that the dopant of Cr atom can reduce the transfer of electrons in Bi_2_Se_3_, as well as the movement of atoms caused by the application of electric field. The band gap of the pure Bi_2_Se_3_ film under electric field is determined by the competition between the electrically tunable charge transfer and Rashba SOC. Significantly, the band gap of Cr-doped Bi_2_Se_3_ film can be engineered by the external electric field, due to the change of charge transfer and magnetism.

## Results

**Figure 1:**
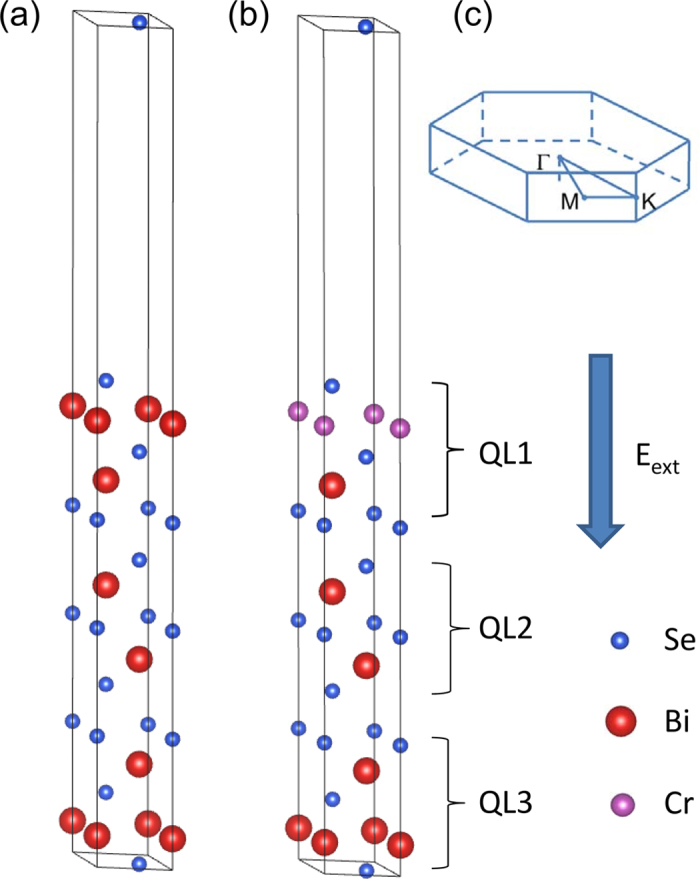
Crystal structure of (**a**) pure and (**b**) Cr-doped 3QL-Bi_2_Se_3_ (001) films. Se, Bi and Cr atoms are denoted by blue, red and purple sphere, respectively. (**c**) Brillouin zone and high symmetry points of the 3QL Bi_2_Se_3_ slab.

Crystalline Bi_2_Se_3_ has a rhombohedral structure and is formed by stacking of weakly coupled quintuple layers (QLs) along its (001) direction. The order of layers in QLs is Se(1)-Bi-Se(2)-Bi-Se(1). It is previously revealed that the thicker the Bi_2_Se_3_ film is, the more sensitive to the external electric field^30^. When the thickness of Bi_2_Se_3_ film becomes larger than 3QL, the band gap drastically reduces as the external electric field applied. Accordingly, the thickness of 3QL where the SSs appears is considered in this study. Figure 1(a) shows the slab structure of pure 3QL Bi_2_Se_3_ film. The lattice constant of a = b = 4.138 Å, c = 28.64 Å taken from the experimental data is chosen for the hexagonal Bi_2_Se_3_ (001) film of 3 QLs. In order to discuss the charge transfer and its response to the external electrical field, QL1, QL2 and QL3 are labeled, representing the upper, middle and lower surfaces, respectively. The outermost Bi atom from the upper surface is substituted by Cr atom, as illustrated in Fig. 1(b). We define the c-axis from QL1 to QL3 as the positive direction of the external electric field (see also Fig. 1). The Brillouin zone and the high symmetry points are also shown in Fig. 1(c).

**Figure 2:**
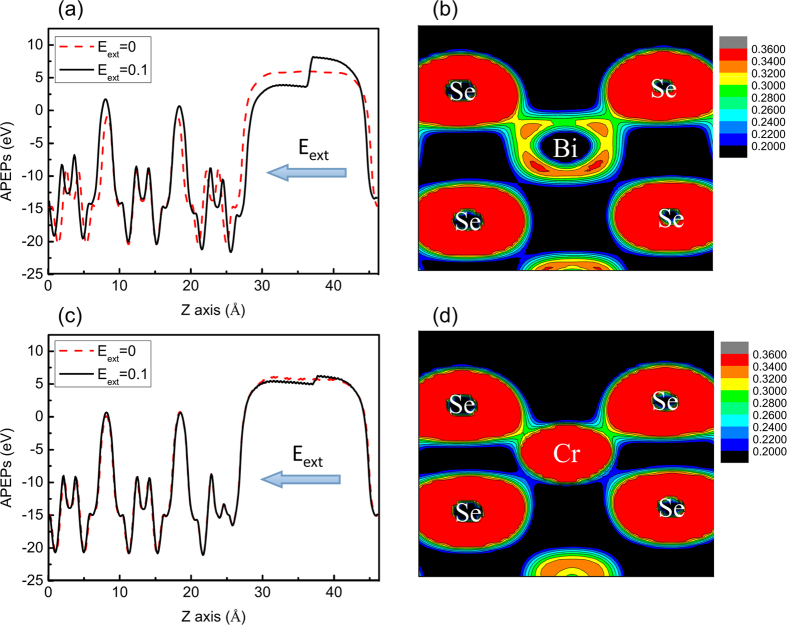
Average planar electrostatic potentials (APEPs) of (**a**) pure and (**c**) Cr doped Bi_2_Se_3_ 3QL film. The APEPs under different external electric fields of 0 and 0.1 V/Å, with its direction shown as the arrow, are marked by (red) dash line and (black) solid line, respectively. Electron density distribution of (**b**) pure and (**d**) Cr doped Bi_2_Se_3_ 3QL film without external electric field are also shown.

Firstly, we study the difference between the atom structures of pure Bi_2_Se_3_ SSs and Cr-doped Bi_2_Se_3_ SSs. It is well known that the radius of Cr atoms is smaller than Bi atoms. Hence the substitution of Cr renders that each layer of the QL1 approaches it. The thickness of QL1 decreases from 7.084 Å to 6.113 Å after doping (see Table 1). This result agrees with the bulk case of Bi_2_Se_3_^22^,^23^. An additional calculation including van der Waals correction shows relaxed interval between QL only slightly changes about 0.046 Å. Subsequently, a series of external electric fields perpendicular to the vacuum layer are applied to the system. Table 1 shows the thickness (*θ*) of each QL and the distance (*d*) between QLs with the electric field of 0 and 0.1 V/Å. We can find that, under the same electric field of 0.1 V/Å, the atom structure of the pure Bi_2_Se_3_ film undergoes an obvious change, while the doped film remains constant. It suggests that the atom structure of the doped system is more robust against the external electric field. As shown in Fig. 2(a) and (c), we calculate the average planar electrostatic potentials (APEPs) of the pure and Cr doped Bi_2_Se_3_ 3QLs film under electric fields of 0 and 0.1 V/Å, respectively. For the pure 3QL-Bi_2_Se_3_ film, the APEPs of two surfaces have the same value of 6.0 eV. As the external electric field is applied (E_ext_ = 0.1 V/Å), the upper and the lower surfaces have different APEPs of 3.6 eV and 8.2 eV, respectively. In the Cr doped case, the APEPs of two surfaces slightly reduce to 5.6 eV when E_ext_ = 0 V/Å. While for the electric field of 0.1 V/Å, the upper and the lower surfaces have less difference than the pure case. The calculated APEPs in Cr doped Bi_2_Se_3_ are 5.0 eV and 6.3 eV for the upper and the lower surfaces, respectively. This result reflects that the upper surface QL1 undergoes an accumulation of electrons, while the lower surface QL3 exports electron when the external electric field is applied toward QL3. The electric potential difference of two surfaces is about 4.6 eV in the pure case, which is larger than that in the doped case with a value of 1.3 eV. It suggests that the doping of Cr in Bi_2_Se_3_ film will reduce the charge transfer caused by external electric field. For the robust atomic structure and the suppression of charge transfer in the Cr doped Bi_2_Se_3_ under the electric field, the main reason can be the movement of each atom in QL1 in ward to the Cr atom. As a result, the covalency of the chemical bonds of QL1 is enhanced, leading to a local redistribution of electrons^22^,^23^,^48^. This result is confirmed by the calculated electron density of pure and Cr doped Bi_2_Se_3_ film, as shown in Fig. 2(b) and (d). Comparing the two figures, we can observe that as Cr atom is introduced, the bonding of Cr with its neighboring Se atoms gets strengthened. Electrons in Cr-doped material become more localized, resulting in the suppression of the charge transfer. This result suggests that the dopant of Cr atom leads to an inhibition of the external electric field.

**Figure 3:**
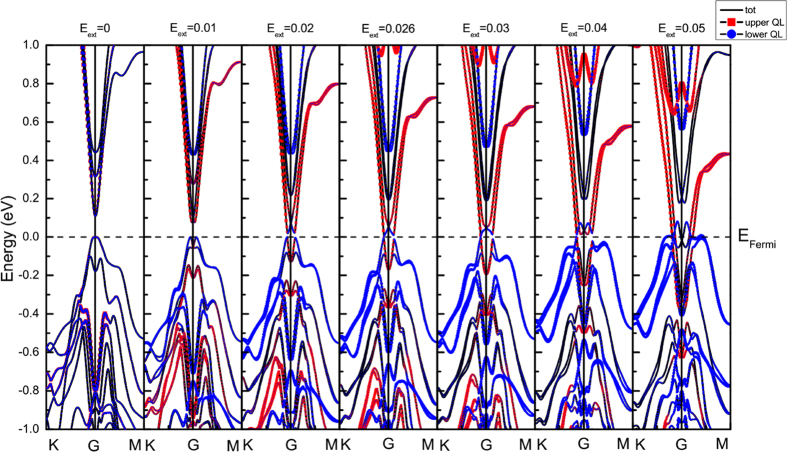
Total band structure (black) for the 3QL-Bi_2_Se_3_ film and the projected band structure for QL1 (upper, red colored) and QL3 (lower, blue colored) under the electric field of 0, 0.01, 0.02, 0.026, 0.03, 0.04 and 0.05 V/Å. The size of the dots denotes the contribution of projected states. The Fermi level is set at the value of 0.

**Figure 4:**
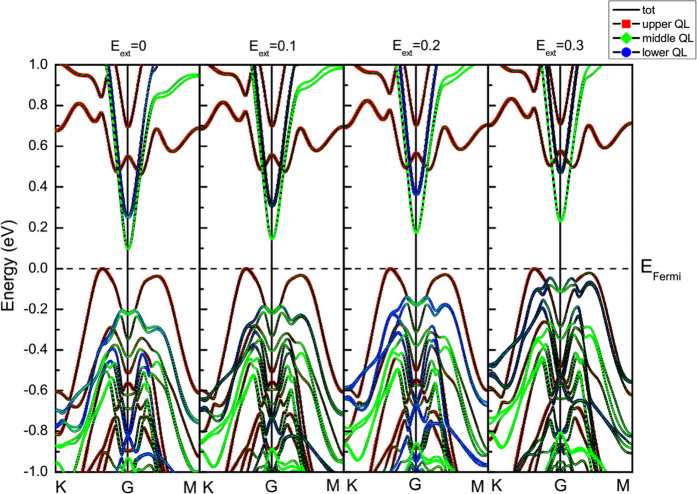
Band structures of the Cr-doped 3QL-Bi_2_Se_3_ film and the projected band structure calculated for QL1 (upper, red colored), QL2 (middle, green colored) and QL3 (lower, blue colored) under external electric fields of 0, 0.1, 0.2 and 0.3 V/Å. The size of the dots denotes the contribution of projected states. The Fermi level is set at the value of 0.

Figure 3 shows the calculated band structures of pure 3QL-Bi_2_Se_3_ film with different external electric fields. When E_ext_ = 0, we get a band gap of 0.111 eV, which is close to the experimental value of 0.138 eV^49^. Due to the symmetrical structure of pure Bi_2_Se_3_, the SSs of QL1 (red) and QL3 (blue) are almost overlapping. Both of the two surfaces contribute to the valence band maximum (VBM) and conduction band minimum (CBM). However, as Cr atom doped, the TRS of Bi_2_Se_3_ film is broken^20^,^22^. The introduction of the magnetic atom results in a removal of the degeneracy, opening a band gap of 0.099 eV in Bi_2_Se_3_, as can be seen in Fig. 4. We can see that, the contribution of the upper and lower QLs in the Cr doped cases no longer overlap without electric field, making it difficult to hybrid between the two surfaces. The major states of VBM are contributed by QL1, which includes the transition metal Cr atom. While the bottom of conduction band consists of the non-degenerate QL2 and QL3 states. Specifically, the CBM is mainly dominated by QL2 as shown in Fig. 4.

**Figure 5:**
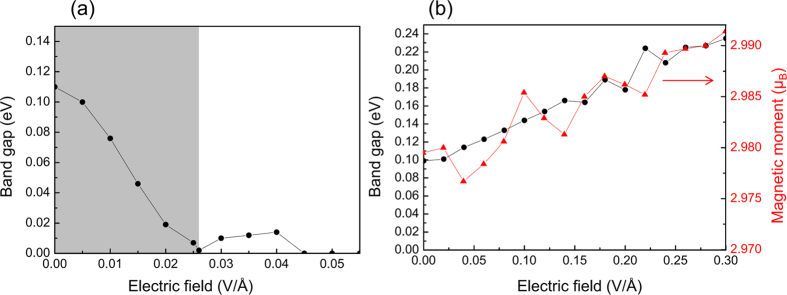
Band gaps (black circles) as a function of the external electric field for (**a**) pure and (**b**) Cr-doped Bi_2_Se_3_ 3QL films. The magnetic moment (red triangles) as a function of the external electric field is also shown in (**b**).

In our calculation, with the increase of electric field from the upper surface to the lower surface, the band gap of pure 3QL-Bi_2_Se_3_ first decreases gradually and close at the field of 0.026 V/Å (see Fig. 3). Furthermore, if the electric field continues to increase, the gap reopens and then closes again, which indicates the band gap of Bi_2_Se_3_ is electrically tunable. The reason is the competition between the electrically tunable charge transfer^28^ and the Rashba SOC. The Rashba SOC is an important ingredient responsible for the nontrivial properties of topological insulator^11^,^12^. In the Bi_2_Se_3_ system, Rashba SOC induces the “M-shape” surface bands, as shown Fig. 3. As the external electric field strengths, the bands of QL1 (red) at VBM rise up while the bands of QL3 (blue) at CBM descend. VBM and CBM turn to touch at a critical gapless point, leading to the band gap closing at E_ext_ = 0.026 V/Å, as shown in Fig. 3. Meanwhile, the bands closing point is not at the Gamma point. As the electric field continues to increase, charge transfer of the system is enhanced. At the same time, the atom positions of Bi_2_Se_3_ are gradually changed, inducing a corresponding strengthening of the SOC inverted bands. Thus, the band gap of Bi_2_Se_3_ will be reopened, as can be clearly seen in Fig. 3 when E_ext_ = 0.04 V/Å. Actually, the interplay between the external electric field and the SOC may induce a quantum phase transition^32^,^45^,^50^ at the critical field of 0.026 V/Å. Finally, with the external electric field strengthens, the charge transfer gradually enhances, making it dominated in the competition, and hence closes the band gap again. Figure 5 clearly shows the trend of band gap as a function of the external electric field. The band gap of pure Bi_2_Se_3_ can be engineered from 0.111 to 0 eV (as the shaded area highlights), where the trend is also predicted by Liu *et al*.^32^ and Wang *et al*.^45^.

We then further investigate the mechanism that electric field directly impact on the band gaps in Cr doped Bi_2_Se_3_ film. Figure 4 shows the calculated band structure for Cr-doped 3QL-Bi_2_Se_3_ film under the electric field of 0, 0.1, 0.2 and 0.3 V/Å, respectively. We can find that Rashba-like band splitting observed in band of pure 3QL-Bi_2_Se_3_ film vanishes in the doping system. Accompanied by the increase of electric field, CBM continuously rises up whereas VBM is pinned below the Fermi level. To clarify this phenomenon, we calculate the decomposed band structures for QL1 (red), QL2 (green) and QL3 (blue) in Fig. 4. It is indicated that, CBM especially the band below 0.4 eV is completely contributed by QL2 and QL3, while VBM is dominated by the Cr-containing QL1. As the electric field increases, the band of QL2 and QL3 apparently move up due to the loss of electrons. Remarkably, the upper surface QL1, theoretically should descend like the pure case, is almost unchanged. As discussed in Fig. 2, this is because the dopant Cr atom can suppress the charge transfer in QL1, making VBM insusceptible under the external electric field. We have also studied the case that Cr atoms substitute Bi atom from a deeper layer. The result shows that the impurity bands of Cr atoms slightly shift down, which has no impact on the band gap. Accordingly, the external electric field will continuously open the band gap of Cr doped Bi_2_Se_3_ film. We conclude the trend in Fig. 5(b). The result shows, the band gap rise from 0.099 eV (under zero field) to 0.144 eV (under the field of 0.1 V/Å). As the external electric field turned up to 0.2 V/Å and 0.3 V/Å, the gap further rises to 0.178 eV and 0.235 eV. It suggests that external electric field is an effective way for engineering the band gap of MTI.

In addition, magnetic order of Cr atom in Bi_2_Se_3_ film is also investigated. Result from a 2 × 2 × 1 supercell Bi_2_Se_3_ surface doped by two Cr atoms shows that ferromagnetic order is preferred (with the total energy lower than antiferromagnetic case by 0.11 eV). This result is consistent with refs 22 and 23. Thus, ferromagnetic order of Cr atom is performed in the study of magnetism. Figure 5(b) shows the calculated magnetic moment as a function of electric field (shown in red line). It shows that with the increase of the electric field, the magnetic moment has the same tendency as the band gap. Under the electric field of 0.3 V/Å, the magnetic moment rises up to 2.991 μ_B_ from 2.979 μ_B_ (at zero electric field). That is, the external electric field will also enhance the magnetic moment due to the change of charge transfer, which is revealed important to the RKKY mechanism by Zhang *et al*.^43^. This result suggests that the magnetic coupling of MTI can also be engineered by external electric field.

## Discussion

**Figure 6:**
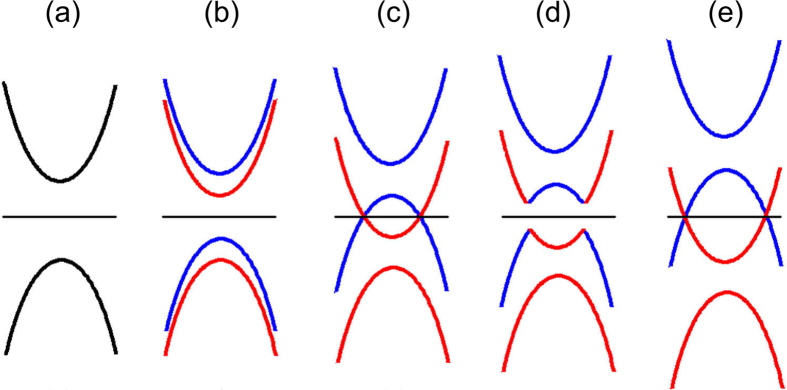
Schematic diagram of the evolution of band structures for pure 3QL-Bi_2_Se_3_ film as the electric field increases. Red and blue curves denote the band structures of QL1 and QL3, respectively.

In order to better understand the influence of external electric field on the system, we describe the schematic diagram of band structure evolution under the increasing electric field. As illustrated in Fig. 6, for pure Bi_2_Se_3_ film, the bands of upper and lower surfaces are degenerate at zero electric field as shown in Fig. 6(a). When the external electric field is applied, the bands of upper (red) and lower (blue) surfaces split away in Fig. 6(b). Due to the direction of electric field originates from the top to the bottom surface (see Fig. 1), the band of upper QL is pushed down while the band of lower QL is lifted up on the contrary, resulting in band gap close, as shown in Fig. 6(c). Figure 6(d) shows that the bands are strongly inversed and a band gap is reopened because of the strong SOC. In this case, the electrically tunable charge transfer^31^ and the Rashba SOC are competing in pure Bi_2_Se_3_. As the electric field continues to increase, the enhanced charge transfer becomes dominant, resulting in the band gap close again, as shown in Fig. 6(e).

**Figure 7:**
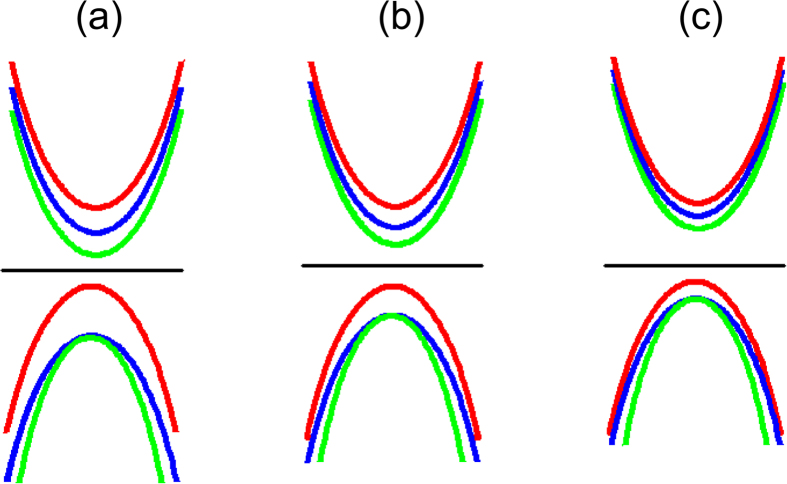
Schematic diagram of the evolution of band structures for Cr-doped 3QL-Bi_2_Se_3_ film as the electric field increases. Red, green and blue curves denote the band structures of QL1, QL2 and QL3, respectively.

For the Cr doped case as shown in Fig. 7, Cr doping leads to the non-degenerate three bands of upper (red), middle (green) and lower (blue) QLs. As the external electric field increases, CBM mainly consisted by QL2 is pushed up, whereas VBM (contributed by the Cr containing QL1) is almost fixed, as can be seen in Fig. 7(b) and (c). Thus, the band gap of Cr doped Bi_2_Se_3_ film will keep enlarging during the increase of the external electric field.

In summary, we have investigated the effects of external electric field on the pure and Cr-doped 3QL-Bi_2_Se_3_ films using first-principles calculations based on DFT. For the pure Bi_2_Se_3_ film, the external electric field will close the band gap at a critical value of 0.026 V/Å. As the electric field continues to increase, the gap may reopen and then close again, which indicates the band gap of Bi_2_Se_3_ is electrically tunable. The competition between electrically tunable charge transfer and Rashba SOC is responsible for this phenomenon. For the Cr doped Bi_2_Se_3_ film, investigation on the APEPs of the two surfaces has suggested that Cr atom doping will suppress the charge transfer, resulting in an inhibition of the external electric field. Calculated band structures indicate that VBM, which is dominated by the Cr atom-containing QL1 surface, is almost fixed under different external electric fields. As a result, the band gap of Cr doped case can be engineered by external electric field from 0.099 to 0.235 eV. Furthermore, magnetic coupling of MTI is revealed can also be manipulated under electric field. Our work provides a new route for engineering the SSs of MTI, promoting the future development of quantum computation and spintronic devices.

## Methods

All the first-principle density functional theory calculation was performed by Vienna *ab initio* simulation package (VASP)^51^,^52^ with the Perdew-Burke-Ernzerhof generalized gradient approximation (GGA-PBE)^53^,^54^. The interaction of ion-electron is described by projected augmented wave (PAW) potentials^55^. To describe the strong electron-electron correlation in partially filled Cr elements, GGA + U calculations with U = 3 eV and J = 0.87 eV are performed^22^,^23^. In particular, SOC is taken into account due to the strong relativistic effect in Bi elements. The cutoff energy for the plane-wave expansion of electron wave function is set to be 300 eV in all the calculations. A 11 × 11 × 1 k-point mesh is adopted for sample the surface Brillouin zone of 3QL Bi_2_Se_3_. We decouple adjacent atomic slabs by using a vacuum layer of 20 Å. All the atoms are allowed to move under the electric field until the forces on each of them are less than 0.02 eV/Å.

## Additional Information

**How to cite this article**: Zhang, J.-M. *et al*. Engineering Topological Surface State of Cr-doped Bi_2_Se_3_ under external electric field. *Sci. Rep.* **7**, 43626; doi: 10.1038/srep43626 (2017).

**Publisher's note:** Springer Nature remains neutral with regard to jurisdictional claims in published maps and institutional affiliations.

## Figures and Tables

**Table 1:** The calculated thickness (θ) of each QL and the distance (d) between QLs in Cr-doped 3QL-Bi_2_Se_3_ films comparing with the pure cases, under the external electric fields of 0 and 0.1 V/Å.

θ/d	Pure Bi_2_Se_3_			Cr doped Bi_2_Se_3_		
	0 V/Å	0.1 V/Å	Δ	0 V/Å	0.1 V/Å	Δ
θ_QL1_ (Å)	7.0844	7.1275	0.0431	6.1137	6.1113	−0.0024
d_QL1-QL2_ (Å)	2.7924	3.3105	0.5181	3.3818	3.3286	−0.0532
θ_QL2_ (Å)	7.0637	7.1064	0.0427	7.1011	7.1157	0.0146
d_QL2-QL3_ (Å)	2.7924	3.3105	0.5181	3.1213	3.2719	0.1506
θ_QL3_ (Å)	7.0844	7.1275	0.0431	7.1079	7.1162	0.0083

Differences (Δ) of the thickness or the distance between 0 and 0.1 V/Å (the values of 0.1 V/Å minus 0 V/Å) are also shown.
